# Mechanisms behind the immediate effects of Roux-en-Y gastric bypass surgery on type 2 diabetes

**DOI:** 10.1186/1742-4682-10-45

**Published:** 2013-07-13

**Authors:** Roland E Allen, Tyler D Hughes, Jia Lerd Ng, Roberto D Ortiz, Michel Abou Ghantous, Othmane Bouhali, Philippe Froguel, Abdelilah Arredouani

**Affiliations:** 1 Department of Physics and Astronomy, Texas A&M University, College Station, TX 77843, USA; 2 Department of Physics, Texas A&M University at Qatar, Education City, PO Box 23874, Doha, Qatar; 3 Genomic Medicine and Systems Biology Research Centre, Qatar Biomedical Research Institute, Doha, Qatar; 4 CNRS-UMR8199, Lille Pasteur Institute, Lille, France; 5 Lille II University, Lille, France; 6 European Genomic Institute for Diabetes, Lille, France; 7 Department of Genomics of Common Disease, School Of Public Health, Hammersmith Hospital, 556 Imperial College, London, UK

## Abstract

**Background:**

The most common bariatric surgery, Roux-en-Y gastric bypass, leads to glycemia normalization in most patients long before there is any appreciable weight loss. This effect is too large to be attributed purely to caloric restriction, so a number of other mechanisms have been proposed. The most popular hypothesis is enhanced production of an incretin, active glucagon-like peptide-1 (GLP-1), in the lower intestine. We therefore set out to test this hypothesis with a model which is simple enough to be robust and credible.

**Method:**

Our method involves (1) setting up a set of time-dependent equations for the concentrations of the most relevant species, (2) considering an “adiabatic” (or quasi-equilibrium) state in which the concentrations are slowly varying compared to reaction rates (and which in the present case is a postprandial state), and (3) solving for the dependent concentrations (of e.g. insulin and glucose) as an independent concentration (of e.g. GLP-1) is varied.

**Results:**

Even in the most favorable scenario, with maximal values for (i) the increase in active GLP-1 concentration and (ii) the effect of GLP-1 on insulin production, enhancement of GLP-1 alone cannot account for the observations. I.e., the largest possible decrease in glucose predicted by the model is smaller than reported decreases, and the model predicts no decrease whatsoever in glucose ×insulin, in contrast to large observed decreases in homeostatic model assessment insulin resistance (HOMA-IR). On the other hand, both effects can be accounted for if the surgery leads to a substantial increase in some substance that opens an alternative insulin-independent pathway for glucose transport into muscle cells, which perhaps uses the same intracellular pool of GLUT-4 that is employed in an established insulin-independent pathway stimulated by muscle contraction during exercise.

**Conclusions:**

Glycemia normalization following Roux-en-Y gastric bypass is undoubtedly caused by a variety of mechanisms, which may include caloric restriction, enhanced GLP-1, and perhaps others proposed in earlier papers on this subject. However, the present results suggest that another possible mechanism should be added to the list of candidates: enhanced production in the lower intestine of a substance which opens an alternative insulin-independent pathway for glucose transport.

## Background

### Type 2 diabetes

Type 2 diabetes (T2D) has reached epidemic proportions worldwide. In 2011, an estimated 366 million people had diabetes, and this number is predicted to rise to 522 million by 2030 [1]. The medical and socioeconomic burdens of the disease and the strains imposed on health-care systems arise from the devastating associated macro- and micro-vascular complications such as nephropathy, hypertension, retinopathy, cardiovascular diseases, and amputations, which make diabetes a major cause of both morbidity and mortality. Cardiovascular morbidity, for example, is 2 to 4 times greater in patients with T2D than in non-diabetic people [2].

T2D develops in adulthood and is generally considered to be a condition marked by insulin resistance and loss of function of insulin-secreting pancreatic beta cells. The exact etiology of T2D remains largely unknown. So far, over 60 genes have been associated with an increased risk for T2D [3]. However, even when pooled, these genes only account for 5-10% of disease risk [4]. Currently, it is widely recognized that obesity is the major independent risk factor for the development of T2D, and that the rise in T2D prevalence worldwide is driven by an increasing frequency of obesity, which, in turn, is driven by a combination of genetic predisposition and interactions with obesogenic environments including high intake of energy-dense food and physical inactivity [5]. Obesity-associated T2D development (diabesity) is due to the excess fat that affects many organs that are involved in glucose homeostasis, including liver and pancreas.

There is a general consensus today that T2D is a lifetime disease and that a medical cure for patients suffering from T2D does not exist. Current medical management of T2D leaves much to be desired, requiring constant vigilance from both patients and physicians. At best, the available medications, when combined with diet and physical activity, are targeted to lower blood glucose and decrease the peripheral insulin resistance associated with T2D. However, medical treatment has had limited success maintaining safe blood glucose levels in patients, as evidenced, for example, by high numbers of diabetic amputations and new onset blindness [6,7]. With minimal success of medical treatment, there is an urgent need for a more permanent cure for the disease that has debilitated so many patients. Currently the only hope for a T2D “cure” (or at least long term remission) is bariatric surgery in very obese patients, which has opened new horizons for understanding the pathophysiology of T2D, and perhaps also hope for new therapies.

### Bariatric surgery

Also called metabolic surgery, this is a form of gastrointestinal surgery that aims at reducing the amount of food intake and/or the absorption of nutrients at the intestinal level. It is, to date, the most successful intervention for the treatment of obesity. In most cases, bariatric surgery achieves a significant and sustained weight loss ranging from 12% to 39% of presurgical body weight or 40-71% excess weight loss (EWL) [8,9].

There exist a dozen bariatric surgery procedures, which fall into the following categories: (i) Restrictive procedure: the aim is to limit the amount of food intake by reducing the size of the stomach. (ii) Malabsorptive procedure: the aim is to limit the absorption of food in the intestinal tract by bypassing a portion of the small intestine to varying degrees. (iii) Combination of both restriction and malabsorption. The four standard bariatric surgery procedures that are currently accepted for weight loss in obese patients are: (1) adjustable gastric banding (AGB), which is solely restrictive; (2) Roux-en-Y gastric bypass (RYGB), which has both restrictive and malabsorptive components; (3) sleeve gastrectomy, which is another solely restrictive procedure used for treatment of morbid obesity; (4) biliopancreatic diversion with duodenal switch (BPDDS), which is a more radical restrictive and malabsorptive procedure [10,11]. Among the four currently accepted procedures, Roux-en-Y gastric bypass (RYGB), where the majority of the stomach and duodenum are bypassed because the stomach is reduced to a small proximal pouch and is then anastomosed to the jejunum [12], and laparoscopic adjustable gastric banding (LAGB), where the stomach is reduced to a small pouch by encircling the upper part of the stomach with a band-like fluid-filled tube, are the most frequently performed worldwide. Sleeve gastrectomy, where the stomach is reduced by 85% by excising the greater curvature and reconstructing a tubularized stomach conduit, is gaining popularity and is widely used for severely obese patients who are high-risk surgery candidates [13].

Bariatric surgery was initially used to induce weight loss in morbidly obese patients. However, it turned out that it also results in an improvement in many obesity related comorbidities including T2D [14]. Today, although curing diabetes cannot yet be considered a goal of bariatric surgery, it may be considered a serendipitous benefit.

### Bariatric surgery induces long-term remission of type 2 diabetes

The last two decades have witnessed the emergence of bariatric surgery as a powerful intervention that, in addition to inducing the drastic and sustained weight loss for which it was initially designed, leads to long-term remission of T2D, decreasing the progression and potentially reversing the effects of diabetes in 40-80% of morbidly obese patients (BMI > 40 kg/m^2^, or BMI > 35 kg/m^2^ with comorbidities) [15-20]. The first report showing a serendipitous improvement or remission of hyperglycemia after gastrectomy was published in the 1950s [21]. However, it was in 1995 that Walter Pories and his colleagues described in a seminal paper a sustained improvement in glycemia control for up to 14 years after gastric bypass surgery in morbidly obese patients with T2D [22]. This astonishingly beneficial effect of bariatric surgery on glucose homeostasis was confirmed by numerous subsequent studies in both humans and animal models [16,18].

It is well documented that weight loss, whatever the means used, improves glycemic control in obese or overweight diabetics, and it is therefore easy to attribute the remarkable return to euglycemia after bariatric surgery to weight loss. However, while weight loss certainly plays a major role in inducing improved glucose homeostasis after metabolic surgery, the striking glycemia normalization after bariatric surgery, as reported by many investigators, is achieved within a few days post-operation, long before any significant weight loss has taken place [22], and many obese diabetic patients are able to decrease, or discontinue, insulin and oral hypoglycemics just days after undergoing surgery [23]. Moreover, there is evidence that even non-obese patients with T2D [24-27] and animal models [28] experience similar anti-diabetic effects without significant weight loss.

This rapid time course and disproportional degree of T2D cure, or at least long-term remission, strongly suggests that resolution of T2D is driven by mechanisms that are surgery-specific and independent of weight loss. Given the immense positive consequences that resolution of T2D will have on the patients’ quality of life and on the diabetes-related expenditure for the patients and for the health-care systems [29], interest in the mechanisms that underlie the remission of T2D has spurred huge interest from the scientific community during the last decade. In fact, understanding the effects of bariatric surgery on T2D would provide important insights into the pathogenesis of type 2 diabetes and allow the development of new procedures, devices, and drugs both for obese and non-obese patients. It is hoped that pharmaceutical mimetics of the underlying mechanisms would potentially offer powerful new medicines for the treatment of T2D without invasive and risky surgery.

### Mechanisms underlying immediate and long-term remission

Despite immense efforts worldwide, the mechanisms behind the glycemic normalization after metabolic surgery remain elusive. It was initially thought that the observed remission of T2D is the obvious result of weight loss, as significant weight loss improves insulin resistance and contributes to diabetes management. However, this hypothesis is challenged by the very rapid adjustment of glycemia reported in many studies, long before any significant weight loss is achieved. Moreover, the positive effect of surgery on glucose tolerance exceeds that achieved after equivalent weight loss via diet and exercise [30] or after conventional medical therapy [18]. Several plausible hypotheses can be articulated to explain the rapid, weight-independent glycemic effects of bariatric surgery, and none of them necessarily precludes the others. In fact, it is likely that the beneficial effect is the result of the involvement of multiple pathways that involve signals to and from different metabolism-related organs including the brain, adipose tissue, pancreas, gastrointestinal tract, liver, kidney, skeletal muscle, and perhaps others. The main suggested hypotheses are listed below. Since there is a vast literature on the effects of bariatric surgery, it should be emphasized that the papers cited here are primarily a representative sample from published work that features the very short-term effects of one specific procedure, namely RYGB.

#### Caloric restriction hypothesis

According to this hypothesis, the remission of T2D after metabolic surgery is due to postoperative caloric restriction. The ability of acute caloric restriction to transiently improve T2D is well known [31,32]. (See, e.g., Figure three of Ref. [32] for 3-7 days of very low calorie dieting.) According to this model, by the time the patients are allowed *ad libitum* eating, they begin to experience the insulin-sensitizing effects of dynamic weight loss from the surgery. Though *prima facie* reasonable, this hypothesis fails to explain why the remission of T2D is far faster after RYGB than AGB, while they both involve perioperative food restriction followed by progressive weight loss [33,34]. This hypothesis also does not explain the superiority of the glycemic control achieved after RYGB versus equivalent weight loss from dieting [10,35].

In general, it appears that extreme caloric restriction (complete fasting or ≤300 kcal per day) can result in strong short-term decreases in plasma glucose and insulin, but that the typical dietary restrictions following RYGB surgery are not sufficient to explain the remarkable improvements that are widely observed.

#### Malabsorption hypothesis

As mentioned above, RYGB has both restrictive and malabsorptive effects, both of which are thought to be significant in achieving long-term weight loss. However, a recent study [36] of patients undergoing long-limb RYGB found that “malabsorption accounted for ≈6% and 11% of the total reduction in combustible energy absorption at 5 and 14 mo, respectively, after this gastric bypass procedure.” In other words, malabsorptive effects are much smaller than caloric restriction effects, and are again insufficent to account for the immediate improvement in glucose levels and insulin sensitivity.

#### Ghrelin hypothesis

Ghrelin is a circulating hormone produced predominantly (90%) by P/D1 cells lining the fundus of the human stomach. It is also produced in small amounts from the pancreas, the intestine, the placenta, the kidney, the pituitary gland, and the hypothalamus. It is an orexigenic hormone that stimulates appetite and food intake. Thus, ghrelin levels increase before meals to signal hunger to the brain, specifically areas of the hypothalamic feeding centers, which express the ghrelin receptors. The latter are also expressed by the insulin-secreting pancreatic beta cells, a key player in glucose metabolism [37]. In addition to its effects on feeding behavior, ghrelin has been implicated in the regulation of glucose homeostasis [38,39]. Thus, the increase of circulating levels of ghrelin by exogenous infusion of the hormone in humans results in a reduction of glucose-induced insulin secretion and therefore glucose disposal [37]. Though the molecular mechanisms by which ghrelin suppresses insulin secretion are not yet well understood, this observation suggests that lower levels of ghrelin may improve the beta cell function.

According to the ghrelin hypothesis, the regulation of ghrelin may be altered by bariatric surgery, and there are indeed studies showing that preprandial ghrelin levels are very low after bariatric surgery. Cummings et al. were the first to report reduced levels of ghrelin post-RYGB compared to pre-RYGB [40], and many subsequent studies have confirmed this observation [41-43]. However, this hypothesis is still very equivocal, as other studies have reported an increase of postprandial ghrelin levels after RYGB, while yet others have reported no change between pre- and post-RYGB [13,19,44]. Different methodologies and study designs might explain these discrepancies, and more investigations are required to fully understand the role of ghrelin in T2D remission after bariatric surgery. Diminished ghrelin secretion would also decrease appetite and food intake, leading to weight loss on a longer time scale than that which is relevant in the present context.

#### Lower intestinal hypothesis

This hypothesis, also called the hindgut hypothesis, is proposed to explain the rapid T2D remission after RYGB and BPD via effects that result from the expedited delivery of nutrients to the lower bowel after an intestinal bypass. It has attracted huge interest because it involves active glucagon-like peptide-1 (GLP-1), an incretin which potentiates insulin secretion and has been shown to increase proliferation and decrease apoptosis of the pancreatic beta cells [45], and which presents great therapeutic potential for the treatment of T2D [46]. According to this hypothesis, the delivery of ingested nutrients to the lower bowel increases GLP-1 release from entero-endocrine L-cells, which are found throughout the small intestine and in high density in the ileum. In fact, a several-fold increase of postprandial active GLP-1 secretion has been reported in a number of studies on patients after RYGB [12,47]. The reported increases range from none to more than 5-fold in the peak value, or more than 10-fold in the area-under-curve value, for the increase in postprandial GLP-1 one week after RYGB, with large error bars (up to about 70%) [48]. There appears to be a clustering of the increases reviewed in Ref. [47] between roughly none and 2-fold or 3-fold, but it is natural that the results will vary with different groups of patients and details of the procedures. We make no attempt in this paper to exclude any published results, or to weight some results more heavily than others. However, it does appear that the case for an increase in GLP-1 levels after RYGB is rather strong. Consistent with elevated postprandial GLP-1 secretion, post-RYGB patients display an increased incretin effect [35].

#### Upper intestinal hypothesis

According to this hypothesis, also called the foregut hypothesis, exclusion of a short segment of proximal small intestine (primarily the duodenum) from contact with ingested nutrients produces direct antidiabetes effects, probably via one or more unidentified duodenal factors that influence glucose homoeostasis. This suggestion is supported by the results for the duodenal-jejunal bypass (DJB) procedure, which maintains the gastric volume intact while bypassing the entire duodenum and the proximal jejunum, and which was tested in several studies that showed an improvement in T2D with no reduction in body weight in animals [49-52] and in obese and non-obese human patients [53,54]. Additional support for this hypothesis comes from the endoluminal duodenal sleeve procedure, where a flexible plastic sleeve is implanted in the upper intestine, causing food to move from the stomach to the beginning of the jejunum without coming in contact with duodenal mucosa. This technique markedly improves glucose tolerance independently of weight loss in rats [55], pigs [56], and humans [57]. This hypothesis is, however, challenged by vertical sleeve gastrectomy, a procedure that does not result in shunting of the duodenum and induces diabetes remission similar to gastric bypass [58-60]. It was also shown very recently that a duodenal bypass procedure without gastric restriction did not resolve T2D [61], in conflict with the foregut hypothesis.

#### Gut microbiota hypothesis

The gut microbiota refers to the billions of microorganisms inhabiting the mammalian gastrointestinal tract. It performs a large number of important roles that define the physiology of the host, such as immune system maturation, the intestinal response to epithelial cell injury, and xenobiotic and energy metabolism. On the other side, it has been directly implicated in the etiopathogenesis of a number of pathological states as diverse as obesity, autism, circulatory disease, inflammatory bowel diseases, and type 1 diabetes [62,63]. The mechanisms through which the microbiota exerts its beneficial or detrimental influences remain largely undefined, but include elaboration of signaling molecules and recognition of bacterial epitopes by both intestinal epithelial and mucosal immune cells [62,63]. Recently, a change in the composition of the gut microbiota after gastric bypass has been reported [64-66], which led some scientists to suggested that the rapid T2D remission after gastric bypass may be partly due to a profound influence of the surgery on the composition of the gut microflora. Mechanisms that may underlie such an effect are, however, poorly known and in need of further exploration.

#### Branched-chain amino acids (BCAAs) hypothesis

The concentrations of branched-chain amino acids (leucine, isoleucine, and valine) were long known to be increased in obese individuals, compared with normal weight-, age-, and sex-matched controls, and the increase was directly correlated with the fasting insulin concentration, a marker of insulin resistance [67]. In a recent prospective study involving individuals followed for 12 years, it has been shown that individuals who had high baseline levels of BCAAs are more prone to develop T2D, which suggests that high concentrations of the BCAAs might be used as a biomarker to aid in diabetes risk assessment [68]. Furthermore, Laferrère et al. [69] reported a significant reduction in circulating total amino acids, especially BCAAs, after bariatric-surgery-induced weight loss, but not after dietary intervention, suggesting that reduction in BCAAs, rather than simply weight loss, may contribute to the rapid improvement in glucose homeostasis and the resolution of T2D seen with gastric bypass surgery. However, a very recent study by Lindqvist and coworkers [70] showed an acute elevation of BCAAs (leucine and valine) after a meal in gastric bypass patients. More work is clearly needed to clarify the role of BCAAs.

## Method

Perhaps the most common starting point in systems biology is a set of first-order ordinary differential equations for the concentrations of biochemical constitutents in various specific regions of an organism. The technique that we introduce here is meant to be applicable to an arbitrarily large set of such equations, but is limited to “adiabatic” (or quasi-equilibrium) states, as defined below.

Let us begin with the general set of equations

(1) $$F_{i}=0\;\text{with}\;i=1,2,\ldots ,N$$

where *F*_*i*_ is a function of the molecular concentrations *x*_*k*_, their derivatives *d*^*n*^*x*_*k*_/*dt*^*n*^, the time *t*, and some set of parameters *r*_*m*_. (Ordinarly *n* = 1, but the elimination of some variables may lead to higher derivatives, as when *dx*/*dt* = *ay* and *dy*/*dt* = *bx* leads to *d*^2^*x*/*dt*^2^ = *abx* after *y* is eliminated). Let *F*_*i*_ → *f*_*i*_ when all the *d*^*n*^*x*_*k*_/*dt*^*n*^ → 0 can be neglected:

(2) $$f_{i}=0$$

in an “adiabatic” state, where the concentrations are changing slowly in comparison to the reaction rates. The knowns in this set of equations are some concentrations (measured or estimated) and some parameters (again measured or estimated – for example, decay rates from half-lifes). The unknowns are the remaining parameters and concentrations. The number of unknowns must be properly matched to the number of equations.

Let us now consider shifts in the concentrations *x*_*k*_ resulting from shifts in the parameters *r*_*m*_. Preservation of the condition *f*_*i*_ = 0 requires that (see below)

(3) $$\frac{\partial f_{i}}{\partial r_{m}}+\underset{k}{\sum }\frac{\partial f_{i}}{\partial x_{k}}\frac{\partial x_{k}}{\partial r_{m}}=0.$$

Eq. (3) has the form

(4) $$\underset{k}{\sum }A_{\text{ik}}V_{k}(m)=B_{i}(m)$$

and this set of linear inhomogeneous algebraic equations can be numerically solved to obtain *∂x*_*k*_/*∂r*_*m*_. Then we can numerically integrate using

(5) $$x_{k}(r+\text{dr})=x_{k}(r)+\underset{m}{\sum }\frac{\partial x_{k}}{\partial r_{m}}dr_{m}$$

where *r* stands for the set of *r*_*m*_.

Eq. (3) follows from *f*_*i*_ = 0 and *f*_*i*_+*df*_*i*_ = 0, with

(6) $$df_{i}=\underset{m}{\sum }\frac{\partial f_{i}}{\partial r_{m}}dr_{m}+\underset{k}{\sum }\frac{\partial f_{i}}{\partial x_{k}}dx_{k}.$$

Here the *r*_*m*_ are varied independently and the *x*_*k*_ vary in response to maintain the steady state, with

(7) $$dx_{k}=\underset{m}{\sum }\frac{\partial x_{k}}{\partial r_{m}}dr_{m}.$$

Eq. (3) follows because the *dr*_*m*_ are independent. More generally, we can replace the *r*_*m*_ by other relevant variables, such as fluxes, changes in environment, or independently-varied concentrations.

The *∂x*_*k*_/*∂r*_*m*_ are obtained at each step in the numerical integration from a numerical solution of Eq. (3). In order for this set of algebraic equations to have a unique solution, we need one equation (labeled by *i*) for each species (labeled by *k*). But this is the natural way to formulate the problem from the beginning.

This paper involved no experimental research or research on humans.

## Results and discussion

### Specific model for immediate effect of bariatric surgery on type 2 diabetes

In constructing a model for the effects of bariatric surgery, with an emphasis on the particularly successful technique of Roux-en-Y gastric bypass, it is important to be aware of the enormous complexity of the biochemical and neural pathways that are affected. However, it is also important to emphasize simplicity, because currently the details of most effects are poorly characterized, and in many cases the reported results are even controversial or contradictory. We are aware of only one previous model [71], which uses 10 equations but focuses on the effect of two incretins, GLP-1 and glucose insulinotropic polypeptide (GIP), since these two constituents are relatively well characterized. Our model is based on a slightly less conservative approach, because it appears that the main relevant effect of GLP-1 in the present context is increased insulin production (with the effects of GIP being smaller and somewhat ambiguous), and that the observed declines in homeostatic model assessment insulin resistance (HOMA-IR) following surgery [72-75] require an additional mechanism. So, in addition to (i) the incretin effect emphasized in Ref. [71], which corresponds to the usual version of the lower intestinal hypothesis, we add the two basic ways in which *insulin resistance* might be immediately ameliorated: (ii) Some set of substances *b* that induce insulin resistance might have their production diminished when the upper part of the digestive tract is bypassed. For example, as mentioned above, there appears to be evidence that insulin resistance may result from branched-chain amino acids [68,69,76,77], as well as from free fatty acids. (Alternative possibilities may include anti-incretins, ghrelin, GIP, and glucagon, which might have a roughly similar effect.) The generic version of this mechanism was also considered in Ref. [71]. (iii) A different version of the lower intestinal hypothesis, in which a postulated substance *a* provides an additional insulin-independent pathway for glucose transport into skeletal muscle cells, and its production is enhanced when digestion is diverted to the lower part of the digestive tract. It has been established that muscle contraction due to exercise opens such an insulin-independent pathway [78-81], and evidence has even been reported for insulin-independent pathways involving nitric oxide [82], some amino acids [83], and bradykinin [84-86]. Of course, in addressing immediate effects of the surgery, we do not include the many other substances that affect appetite etc. but do not appear to be of major importance for remission after only a few days. For simplicity, we are regarding caloric restriction, ghrelin effects, and improvements in beta cell function as primarily longer-term than a few days, although one should bear in mind that these effects can be added to those explicitly included in the model. Finally, we mention that the gut microbiota hypothesis may be consistent with any of the effects in the model.

To summarize the above paragraph, we include three mechanisms for producing a decrease in glucose levels and also insulin resistance: The first is an increase in the production of incretins (primarily GLP-1). The second is a decrease in the production of substances, labeled *b*, which contribute to insulin resistance. The third is an increase in a substance *a* which provides an additional pathway for glucose to enter cells through the plasma membrane.

### Time-dependent equations

Now let us write down the equations describing the time evolution of the five molecular concentrations *x*_*k*_ which are regarded as most central in the regulation of the plasma glucose level, with *x*_*G*_ representing glucose, *x*_*I*_ insulin, *x*_*I*_ incretins (with emphasis on GLP-1), *x*_*b*_ substances which increase insulin resistance, and *x*_*a*_ a substance which opens an insulin-independent pathway for glucose transport. The model is defined by

(8) $$\begin{array}{l} \frac{dx_{G}}{\text{dt}}=R_{G}-r_{\text{GI}}x_{G}x_{I}e^{-\alpha_{b}x_{b}}-r_{\text{Ga}}x_{G}x_{a}-r_{G}x_{G} \end{array}$$

(9) $$\begin{array}{l} \frac{dx_{I}}{\text{dt}}=r_{\text{IG}}x_{G}+r_{\text{Ii}}x_{G}x_{i}-r_{I}x_{I} \end{array}$$

(10) $$\begin{array}{l} \frac{dx_{i}}{\text{dt}}=rR_{i}-r_{i}x_{i} \end{array}$$

(11) $$\begin{array}{l} \frac{dx_{a}}{\text{dt}}=rR_{a}-r_{a}x_{a} \end{array}$$

(12) $$\begin{array}{l} \frac{dx_{b}}{\text{dt}}=\left(r_{\text{max}}-r\right)\;R_{b}-r_{b}x_{b}. \end{array}$$

In Eq. (8), *R*_*G*_ is the rate at which glucose is received by the plasma from digestion; *r*_*GI*_ gives the rate at which insulin stimulates glucose transport in the absence of the “bad” substances *b*; $e^{-\alpha_{b}x_{b}}$ is a factor representing the contribution of *b* to insulin resistance; and *r*_*Ga*_ determines the rate at which the postulated “good” substance *a* stimulates glucose transport via an alternative pathway. (*R*_*G*_ is actually the rate at which glucose is made available to the cells which normally absorb glucose via an insulin-dependent pathway, and which are therefore relevant in the present context – e.g., skeletal muscle cells.) In Eqs. (8)-(12), the final terms represent “natural disappearance”. (The degradation of incretins by dipeptidyl-peptidase 4 (DPP4) is thus not explicitly exhibited.) In Eq. (9), *r*_*IG*_ gives the rate at which glucose stimulates insulin production (in the pancreas), and *r*_*Ii*_ represents the enhancement by incretins. In Eq. (10), the production of incretins (stimulated by glucose in the intestine) is increased by a factor *r*, with a pre-surgery value *r* = 1 and a post-surgery value *r* = *r*_max_. Finally, in Eqs. (11) and (12), *rR*_*a*_ and (*r*_max_−*r*)*R*_*b*_ are respectively the input of substances *a* and *b* from digestion, with the same scaling assumed as for the incretins.

### Equations in an “adiabatic” postprandial state

Let us now consider an “adiabatic” state in which the concentrations are slowly varying (e.g. on a time scale ∼ hours, with a time scale for relevant reactions typically ∼ minutes). In the present context, we are concerned with postprandial states, since these are the most important in relation to glucose homeostasis. (In the fasting state, with glucose secreted from the liver rather than intestine, there would be essentially no production of incretins, and Eqs. (10)-(12) would be invalid.) There is, of course, a continuous evolution up to and down from the peak concentrations of glucose, insulin, GLP-1, etc. following ingestion, but in the model these concentrations are taken to evolve together. The scaled values which are used here may therefore apply at any point (over a period of several hours), and when integrated over time they also apply to the “area under curve” or average values of the concentrations.

With time derivatives neglected, the linear equations are trivially solved:

(13) $$\begin{array}{l} x_{i}=r\frac{R_{i}}{r_{i}} \end{array}$$

(14) $$\begin{array}{l} x_{a}=r\frac{R_{a}}{r_{a}} \end{array}$$

(15) $$\begin{array}{l} x_{b}=\left(r_{\text{max}}-r\right)\;\frac{R_{b}}{r_{b}}. \end{array}$$

(16) $$\begin{array}{l} x_{I}=\frac{1}{r_{I}}\left(r_{\text{IG}}+r_{\text{Ii}}r\;\frac{R_{i}}{r_{i}}\right)x_{G}. \end{array}$$

Substitution into the equation for *x*_*G*_ then results in a quadratic equation. One can easily obtain the explicit solution for *x*_*G*_ in the present case, but it is clear that this approach does not readily generalize to problems where one can have a very large number of coupled equations. This fact is what motivates the general method described in the preceding section, in which the parameters *r*_*m*_ are treated mathematically as continuous variables. According to Eq. (3), the solution for the derivatives *∂x*_*k*_/*∂r*_*m*_ is a linear algebraic problem. This problem, and the subsequent integration with respect to the *r*_*m*_, can then be solved with standard numerical methods. An additional advantage is that some parameters may disappear when the derivatives are taken – for example, *R*_*G*_ in Eq. (8).

### Effect of surgery treated as a continuous variable

Using this approach (with *r* regarded as a continuous variable) one obtains

(17) $$\begin{array}{l} \frac{dx_{i}}{\text{dr}}=\frac{R_{i}}{r_{i}} \end{array}$$

(18) $$\begin{array}{l} \frac{dx_{a}}{\text{dr}}=\frac{R_{a}}{r_{a}} \end{array}$$

(19) $$\begin{array}{l} \frac{dx_{b}}{\text{dr}}=-\frac{R_{b}}{r_{b}} \end{array}$$

(20) $$\begin{array}{l} \frac{dx_{I}}{\text{dr}}=\left(\frac{r_{\text{IG}}}{r_{I}}+\frac{r_{\text{Ii}}\;R_{i}}{r_{I}r_{i}}r\right)\frac{dx_{G}}{\text{dr}}+\frac{r_{\text{Ii}}R_{i}}{r_{I}r_{i}}x_{G}. \end{array}$$

These are mathematical equations, but one can imagine a thought experiment in which these equations, and those below for *x*_*G*_, describe the rates at which concentrations of chemical species change as more and more of the upper digestive tract is bypassed. Applying *d*/*dr* to the condition (from Eq. (8))

(21) $$\begin{array}{ll} -R_{G}+r_{\text{GI}}x_{G}x_{I}e^{-\alpha_{b}x_{b}}+r_{\text{Ga}}x_{G}x_{a}+r_{G}x_{G}=0 & \; \end{array}$$

then leads finally to the following equation for glucose alone:

(22) $$\begin{array}{ll} \frac{dx_{G}}{\text{dr}}=-\frac{a_{i}e^{\alpha r}x_{G}^{2}+\alpha \left(a_{I}+a_{i}r\right)e^{\alpha r}x_{G}^{2}+a_{a}x_{G}}{2\left(a_{I}+a_{i}r\right)e^{\alpha r}x_{G}+a_{a}r+r_{G}} & \; \end{array}$$

where

(23) $$\begin{array}{rl} a_{I}=\frac{r_{\text{GI}}r_{\text{IG}}}{r_{I}}e^{-\alpha r_{\text{max}}}\;, & \;a_{i}=\frac{r_{\text{GI}}r_{\text{Ii}}R_{i}}{r_{I}r_{i}}e^{-\alpha r_{\text{max}}}\; \end{array}$$

(24) $$\begin{array}{rl} \alpha =\alpha_{b}\frac{R_{b}}{r_{b}}\;, & \;a_{a}=r_{\text{Ga}}\frac{R_{a}}{r_{a}}. \end{array}$$

Here *a*_*I*_, *a*_*I*_, *α*, and *a*_1_ respectively represent the strengths of the glucose-insulin-glucose interaction (i.e., insulin-stimulated glucose transport into cells), glucose-insulin-incretin-glucose interaction (i.e., the enhancement by incretins), insulin resistance due to substances *b*, and direct glucose transport due to substance *a*. We can scale these strengths by dividing numerator and denominator by the strength *a*_*I*_ of the usual insulin mechanism:

(25) $$\begin{array}{ll} \frac{dx_{G}}{\text{dr}}=-\frac{c_{i}e^{\alpha r\;}x_{G}^{2}+\alpha \left(1+c_{i}\;r\right)e^{\alpha r\;}x_{G}^{2}+c_{a}x_{G}}{2\left(1+c_{i}r\right)e^{\alpha r\;}x_{G}+c_{a}r+c_{G}} & \; \end{array}$$

with

(26) $$\begin{array}{ll} c_{i}=a_{i}/a_{I}=\frac{r_{\text{Ii}}R_{i}}{r_{\text{IG}}r_{i}}\;,\;c_{a}=a_{a}/a_{I}\;,\;c_{G}=r_{G}/a_{I}\;. & \; \end{array}$$

We also scale the glucose concentration (along with the parameters) so that *x*_*G*_ = 1 for *r* = 1.

### Test of incretin hypothesis

The most common hypothesis to explain type 2 diabetes remission immediately after surgery is that it is due solely to a large enhancement of (active) GLP-1 production in the lower intestine, with a nice review of the many studies in this area by Rhee et al. [47].

We now set out to test this hypothesis. The measured enhancements of active GLP-1 range from none (or a slight decrease) to more than 10-fold [47,48], with the majority of reported values being well below 10, and in fact ≲ 2 or 3 [47], so we will show the results for

(27) 1≤r≤10.

Of course, one expects variations among different groups of patients, and with different postoperative restrictions. It should also be mentioned that there are different forms of GLP-1, and that the half-life of active GLP-1 in circulation is less than 2 minutes.

The normal contribution of incretins (mainly GLP-1) to insulin secretion is usually considered to be of the order of 50−70% [87,88]. As can be seen from Eqs. (9), (13) (with *r* = 1), (23), and (26),

(28) $$1\leq c_{i}\leq 2$$

corresponds to the incretin contribution to insulin secretion lying between 50% and 67%. But we will also include the case of 100% (implying that there would be no insulin production whatsoever without the assistance of incretins), which is given by *a*_*I*_ = 0 in Eq. (22), or

(29) $$1+c_{i}\;r\to c_{i}\;r$$

in Eq. (25).

**Figure 1:**
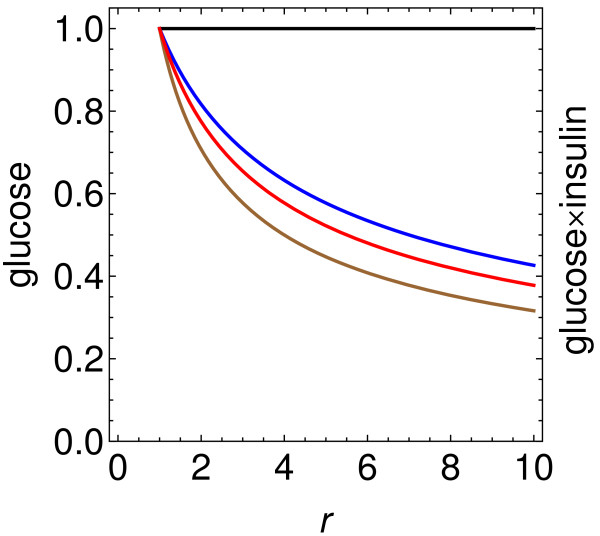
**Effect of incretin concentration alone.** Two tests of the hypothesis that an increase in incretin concentration alone can explain the fall in glucose level and homeostatic model assessment insulin resistance (HOMA-IR) immediately after surgery. The three lower curves show the scaled glucose concentration *x*_*G*_ as a function of the factor *r* by which active incretins are increased. (Reported values of *r* range from 1 to more than 10, with most ≲ 2 or 3.) These curves correspond to three assumptions regarding the incretin contribution to insulin production: 50% for the top curve, 67% for the middle curve, and 100% for the bottom curve. Even in the most favorable scenarios, the decrease is insufficient to explain all the observations. The horizontal line at the top is the scaled product glucose × insulin, or *x*_*G*_ × *x*_*I*_, which is a measure of insulin resistance analogous to HOMA-IR. As found in Eq. (33), it is constant for all scenarios – i.e., for all values of *r* and all percentages for the incretin contribution. In other words, the incretin mechanism alone predicts no decrease whatsoever in this quantity. Many observations, on the other hand, show a very substantial drop in HOMA-IR immediately or very soon after surgery.

In Figure 1, results are shown for the above hypothesis, that the immediate effects of surgery are due entirely to increased incretin production, and for essentially the full range of values consistent with experiment. The three lower curves in Figure 1 show the decline in glucose concentration for up to a 10-fold increase in incretin production, and the horizontal line at the top shows the corresponding result for glucose × insulin, or *x*_*G*_ × *x*_*I*_, which is a measure of insulin resistance analogous to homeostatic model assessment insulin resistance (HOMA-IR) measured in the fasting state. The higher two of the descending curves correspond to *c*_*i*_ = 1 and 2 in Eq. (25), with no contribution from substances other than incretins:

(30) $$\frac{dx_{G}}{\text{dr}}=-\frac{c_{i}x_{G}}{2\left(1+c_{i}r\right)}\;.$$

The lowest curve corresponds to the upper bound that 100% of insulin secretion is due to incretins, and it will also be discussed below.

Here we have omitted the term *c*_*G*_ in the denominator, which correponds to the “natural disappearance” of glucose through excretion. This means that we are *overestimating* the decline in glucose as a function of *r*, since omission of *c*_*G*_ in the denominator increases the magnitude of the negative derivative. The lowest curve in the figures are therefore extreme lower bounds on how much the glucose level and the glucose ×insulin can decline if only incretins are involved, and if 10 is taken to be the upper bound on *r*.

The solution to Eq. (30) is

(31) $$x_{G}={\left(\frac{1+c_{i}}{1+c_{i}r}\right)}^{1/2}.$$

It can be substituted into Eq. (16), which becomes

(32) $$x_{I}=\frac{1+c_{i}r}{1+c_{i}}x_{G}$$

after the insulin concentration (along with the parameters) is scaled to make *x*_*I*_ = 1 for *r* = 1. It follows that

(33) $$\text{glucose}\times \text{insulin}=\frac{1+c_{i}r}{1+c_{i}}x_{G}^{2}=1.$$

Therefore, in every scenario for glucose reduction being produced entirely by incretins, there is no drop whatsoever in glucose × insulin, a quantity which is the analog in a postprandial state of HOMA-IR. The observations show, on the other hand, that HOMA-IR typically drops immediately after surgery [72-75], e.g. by 50% after one week in both obese subjects with T2D and matched subjects with normal glucose tolerance [48].

Again, the lowest curve in Figure 1 corresponds to the upper bound that incretins account for 100% of insulin secretion. In this case, according to Eq. (29), Eq. (31) reduces to

(34) $$x_{G}=\frac{1}{r^{1/2}}.$$

The most extreme limit of possible scenarios thus gives a drop in glucose concentration of about $1/\sqrt{10}\approx 0.32$, but more commonly reported values of *r* and *c*_*i*_ give drops of about 0.7−1.0, as can be seen in Figure 1. On the other hand, some observations show much stronger decreases soon after surgery – e.g., a drop by a factor of 0.31 (from 495 mg/dL to 153 mg/dL) in 14 days [10]. The expected long-term recovery of *β*-cells seems unlikely to produce such a large decrease so quickly, and in other cases there are large drops in as little as 3 days.

There are two qualitative reasons for the above results: (i) The incretins are effective in increasing the insulin concentration, but not the insulin sensitivity of the cells. (ii) The effect of the incretins is second-order in the glucose concentration, as can be seen in Eq. (25). In other words, as the glucose concentration falls, the insulin concentration also falls, with the rate of glucose absorption being proportional to the product of these concentrations. On the other hand, the postulated substance *a* would have an effect that is first-order, because it directly stimulates glucose transport without insulin as an intermediary.

In summary, our results indicate that the most plausible values for an increase in GLP-1 can largely, but not completely, explain the observed beneficial changes immediately after surgery. Let us now turn to the other possibilities, considering each separately.

### Test of branched-chain amino acids hypothesis (and other foregut hypotheses)

First consider the effect of only relieving the extra insulin resistance due to substances *b*, by setting *c*_*i*_ = *c*_*a*_ = 0 (and *c*_*G*_ = 0) in Eq. (25), so that *dx*_*G*_/*dr* = −*αx*_*G*_/2 and

(35) $$x_{G}=e^{-\alpha r/2}.$$

If *αr*_max_/2 is substantial, then the glucose concentration will undergo a substantial drop. This fact lends some credibility to the branched-chain amino acids hypothesis discussed above [68,69,76,77], even though insulin resistance is more commonly attributed to the release of lipids from adipose tissue.

These results actually have more general validity, since this same model can be applied to any factor from the stomach or upper intestine (duodenum and jejunum) that induces insulin resistance.

### Possibility of alternative insulin-independent pathway for transport of glucose into muscle cells

Finally consider the effect of increasing only the influence of the postulated substance *a*, which opens an alternative pathway, by setting *c*_*i*_ = *α* = 0 (and again *c*_*G*_ = 0). Eq. (25) becomes

(36) $$\frac{dx_{G}}{\text{dr}}=-\frac{c_{a}x_{G}}{2x_{G}+c_{a}r}\;.$$

**Figure 2:**
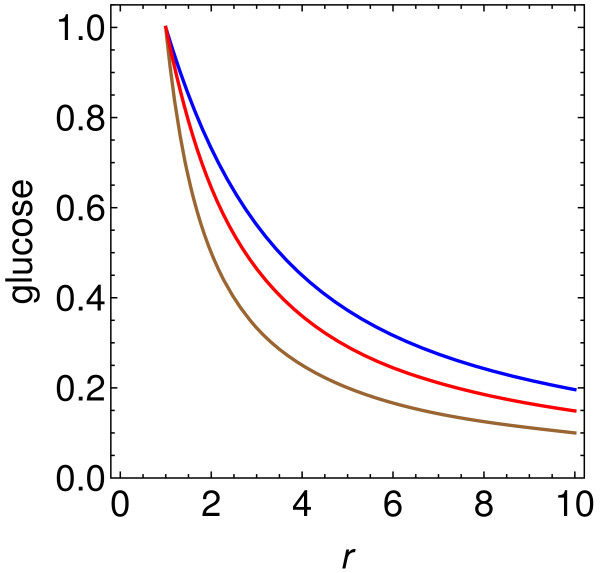
**Effect of alternative insulin-independent pathway.** Glucose concentration *x*_*G*_ as a function of the increase *r* in a substance *a* which opens an alternative insulin-independent pathway for glucose absorption (by the cells which are relevant in the present context). The top and middle curves are respectively for *c*_*a*_ = 1 and 2, where *c*_*a*_ is the strength of this alternative pathway relative to the normal insulin-dependent pathway in a patient with strong insulin resistance. The bottom curve represents the limit of extreme insulin resistance. The scaled product glucose × insulin is given by exactly these same curves, since the insulin level is constant in this case. If the present mechanism and that of Figure 1 are both operative, there is, of course, an even larger drop in glucose level, and also a substantial drop in glucose × insulin. This product, in a postprandial state, is a measure of insulin resistance analogous to HOMA-IR – which is the same product measured in the fasting state.

The results are shown in Figure 2. Since the insulin level does not change in this case, the same curve describes glucose × insulin. In the limiting case of extreme insulin resistance, with *a*_*I*_ = 0 in Eq. (22), Eq. (25) reduces to

(37) $$\frac{dx_{G}}{\text{dr}}=-\frac{x_{G}}{r}$$

so

(38) $$x_{G}=\frac{1}{r}.$$

A substance opening a new pathway could thus have a strong effect if it were produced in appreciable abundance.

In the limiting cases, the 1/*r* decrease of Eq. (38), in both glucose level and glucose × insulin, represents a first-order effect. On the other hand, the 1/*r*^1/2^ decrease of Eq. (34) in glucose level, with no drop of glucose × insulin in Eq. (33), represents a second-order effect, as defined below Eq. (34). This is a simple way of understanding why an insulin-independent pathway would be so effective in reducing both glucose and glucose × insulin.

## Conclusions

Our general method was employed in a simple model of the response of plasma glucose concentration to bariatric surgery (with the paradigm being Roux-en-Y gastric bypass). This model includes three mechanisms that might be responsible for the remarkable positive effect observed for most patients immediately following surgery, before any appreciable weight loss. The first mechanism is the one which is currently the most widely embraced: increased production of incretins (mainly GLP-1). We performed calculations up to and including the most favorable scenario, in which there is about a 10-fold increase in the incretins and the incretins account for 100% of insulin secretion. The results, shown in Figure 1, indicate that the most plausible values for an increase in GLP-1 alone cannot fully account for the decreases in glucose level which have been reported, or the large and rapid observed decreases in HOMA-IR.

In other words, we find that GLP-1 can largely, but not completely, explain the observed beneficial changes immediately after surgery.

Another possible mechanism, involving insulin resistance which is diminished when the stomach and upper intestine are bypassed, could be effective if this were indeed the main cause of type 2 diabetes in the present context. However, for obese patients undergoing bariatric surgery, the cause of insulin resistance is more commonly thought to be the release of fatty acids from fatty tissue, which will decrease only after appreciable weight loss.

This leaves the possibility that diversion of food to the lower intestine results in the production of a substance which opens an alternative insulin-independent pathway for transport of glucose into cells. As mentioned above, it has been established that exercise opens an insulin-independent pathway [78-81], involving an alternative pool of intracellular GLUT-4 which activates glucose transport through the cell membrane, and it has been argued that there are additional insulin-independent pathways involving nitric oxide [82], some amino acids [83], and bradykinin [84-86], so there are precedents for such a mechanism.

The results of Figure 2 demonstrate that this would be a quite robust mechanism, which would produce large decreases in both plasma glucose and the product glucose × insulin, which provides a measure of insulin resistance analogous to HOMA-IR. If such a substance could be detected, it might, of course, be relevant to pharmaceutical approaches.

In summary, the present results suggest that another possible mechanism should be added to the current list of potential explanations for immediate glycemia normalization following Roux-en-Y gastric bypass surgery: enhanced production in the lower intestine of a substance which opens an alternative insulin-independent pathway for glucose transport.

## Competing interests

The authors declare that they have no competing interests.

## Authors’ contributions

AA proposed the project, surveyed the literature, and wrote the Background section. RA further surveyed the literature, devised the method, formulated the model, solved the equations, and wrote the remainder of the paper. TH, JLN, and RO participated in regular discussions and provided input for formulation of the model and the biomedical context. MAG and OB verified all the mathematics. MAG additionally created the figures and made suggestions regarding the biomedical context. PF contributed to discussion and revision of the paper. All authors read and approved the final manuscript.
